# Towards Personalized Therapy of Aortic Stenosis

**DOI:** 10.3390/jpm11121292

**Published:** 2021-12-03

**Authors:** Piotr Mazur, Magdalena Kopytek, Michał Ząbczyk, Anetta Undas, Joanna Natorska

**Affiliations:** 1 Department of Cardiovascular Surgery, Mayo Clinic, Rochester, MN 55902, USA; piotr.k.mazur@gmail.com; 2 Institute of Cardiology, Jagiellonian University Medical College, 80 Pradnicka St, 31-202 Kraków, Poland; m.kopytek@szpitaljp2.krakow.pl (M.K.); michalzabczyk@op.pl (M.Z.); anetta.undas@uj.edu.pl (A.U.); 3 Center for Research and Medical Technologies, John Paul II Hospital, 31-202 Kraków, Poland

**Keywords:** calcific aortic stenosis, non-vitamin K antagonist oral anticoagulants, advanced glycation end products, tissue inhibitors of matrix metalloproteinases, inflammation, calcification, lipid lowering therapy

## Abstract

Calcific aortic stenosis (CAS) is the most common cause of acquired valvular heart disease in adults with no available pharmacological treatment to inhibit the disease progression to date. This review provides an up-to-date overview of current knowledge of molecular mechanisms underlying CAS pathobiology and the related treatment pathways. Particular attention is paid to current randomized trials investigating medical treatment of CAS, including strategies based on lipid-lowering and antihypertensive therapies, phosphate and calcium metabolism, and novel therapeutic targets such as valvular oxidative stress, coagulation proteins, matrix metalloproteinases, and accumulation of advanced glycation end products.

## 1. Introduction

Calcific aortic stenosis (CAS) is the most common cause of acquired valvular heart disease in the adult population of the Western world, with no available pharmacological treatment to inhibit the disease progression to date. CAS is characterized by the progressive leaflet thickening and fibro-calcification of tissue. The clinical manifestation of the disease results from the subsequent obstruction of blood flow from the left ventricle to the aorta [1]. Symptomatic severe CAS is associated with poor prognosis, and to date surgical aortic valve replacement (SAVR) or transcatheter aortic valve implantation (TAVI) are the only available treatment options [2,3]. While the outcomes of both procedures are excellent in select groups of patients, the invasive management merely relieves the symptoms at the very late stage of the disease and does not address the CAS pathophysiology. It is estimated that about 4.5 million cases of hemodynamically significant CAS will be present worldwide by the year 2030, with no means to address the problem pharmacologically [4].

The concept of CAS as an atherosclerotic process is supported by epidemiologic studies showing that its development is associated with cardiovascular risk factors, such as arterial hypertension, hypercholesterolemia, diabetes mellitus (DM), male gender, cigarette smoking, or older age [5,6]. CAS development is spread out in time, and its progression is a complex process associated with activation of mechanisms at the molecular, cellular, and tissue levels [7]. Currently, CAS is considered an active inflammatory process that occurs in response to damage of valvular endothelium and an influx of low-density lipoproteins (LDL) and monocytes. This leads to the activation of valve interstitial cells (VICs). Many of them differentiate into fibroblasts with an osteoblastic phenotype, which are associated with valvular calcification. Due to high morbidity and mortality, the need for interventional treatment and growing medical costs of CAS management, pharmacological therapies targeted at valvular calcification at early stages of CAS are extensively studied. However, none of them have been approved so far. In this review we present the contemporary literature evidence on molecular pathways and their potential therapeutic implications for this disease.

## 2. Pathophysiology of Aortic Valve Calcification

The architecture of the aortic valve is highly conservative to withstand a wide range of stresses during the cardiac cycle, both hemodynamic and mechanical [8]. A normal aortic valve is composed of three tissue layers, each one with a specific arrangement. On the aortic side of leaflet is the fibrosa layer, rich in fibroblasts and collagen fibers arranged circumferentially. On the ventricular side of the leaflet lies the ventricularis, abundant in elastin fibers aligned in a radial direction, providing flexibility to the aortic valve. Between these two layers is the spongiosa, which is composed of loosely arranged layer of connective tissue containing fibroblasts, mesenchymal cells and a mucopolysaccharide-rich matrix. Within aortic valve leaflets, two populations of cells can be distinguished: valve endothelial cells (VECs) and VICs, which are responsible for maintaining of the valvular structure and function. VECs are present on both sides of the cusp, while VICs lying within the extracellular matrix (ECM) fill the body of the aortic valve. VICs are the predominant cells within the aortic valves and as a heterogeneous population include fibroblasts, myofibroblasts, and smooth muscle cells [9]. Liu et al. [10] have discovered five phenotypes of VICs such as: embryonic progenitor endothelial/mesenchymal cells, quiescent VICs (qVICs), activated VICs (aVICs), progenitor VICs (pVICs), and osteoblastic VICs (obVICs). Each of these phenotypes represents specific features associated with valve remodeling and repair. Recently, the new subpopulation of VICs, double positive for CD34 and platelet-derived growth factor receptor α (PDGFRα), has been shown to exist within normal aortic valves, possibly involved in the maintenance of the local microenvironment counteracting a pathological valve remodeling [11]. The reduced number of such VICs could be also associated with increasing risk of CAS development in older age [11]. Persistent activation of VICs plays a pivotal role as a trigger of valvular calcification [10]. Activated VICs are responsive to typical osteogenic mediators such as members of the transforming growth factor-β (TGF-β) superfamily, and bone morphogenetic proteins (BMPs) [12,13]. Aortic leaflets calcification is driven by BMPs that activate Smad1/5/8 and Wnt/β-catenin signaling pathways. Smad1/5/8 activation leads to up-regulation of master osteoblast transcription factor runt-related transcription factor 2/core binding factor 1 (Runx2/Cbfα1), which in turn enhances the expression of factors directly associated with calcification and osteoblast differentiation [12,13]. Besides VICs, macrophages, lymphocytes, cardiac chondrocytes, mast cells, and neutrophils are involved in valvular inflammation, leading to subsequent calcium nodule formation [14,15,16,17].

### 2.1. Lipid-Lowering Therapy

Large retrospective studies including SALTIRE (Scottish Aortic Stenosis and Lipid Lowering Trial) [18], SEAS (The Simvastatin and Ezetimibe in Aortic Stenosis) [19], ASTRONOMER (the Aortic Stenosis Progression Observation: Measuring the Effects of Rosuvastatin) [20], and the PROCAS (Effects of Rosuvastatin on Progression of Stenosis in Adult Patients With Congenital Aortic Stenosis) [21] have demonstrated that intensive treatment statins does not inhibit progression of CAS [22]. Therefore, the current guidelines do not endorse the use of statins for the treatment of CAS [3].

However, a secondary analysis of the data from the ASTRONOMER trial showed that elevation of lipoprotein(a) [Lp(a)] and oxidized phospholipids levels is associated with a linear progression of valvular calcification in mild-to-moderate CAS patients [22]. A proprotein convertase subtilisin/kexin type 9 (PCSK9), an enzyme produced in the liver influencing the expression levels of the LDL receptor at the surface of hepatic cells, influences the LDL cholesterol levels and its mutations have been associated with derangements in lipid metabolism [23]. Langsted et al. [24] have reported that the PCSK9 loss-of-function mutation was associated with lower serum levels of LDL and Lp(a), and reduced CAS risk. Not surprisingly, PCSK9 inhibitors (designed as monoclonal antibodies: alirocumab and evolocumab) are currently tested as a pharmacological option to retard CAS progression [25]. The exploratory analysis of the FOURIER (Further Cardiovascular Outcomes Research with PCSK9 Inhibition in Subjects With Elevated Risk) randomized clinical trial revealed nearly 50% lower incidence of CAS after a median 2.2 (1.8–2.5) years of treatment with evolocumab compared to placebo [26]. Remarkably, Perrot et al. [27] have recently shown an increased expression of PCSK9 within the stenotic aortic valves, and about 50% reduced VICs calcium levels measured in vitro after addition of anti-PCSK9 antibody.

Lp(a) has been a promising therapeutic target for a variety of therapies. A significant reduction of both Lp(a) (−25%) and LDL cholesterol (−41%), along with an increase in high-density lipoprotein (HDL) cholesterol levels (+104%), has been reported in atherosclerotic patients treated with anacetrapib, a cholesteryl ester transfer protein (CETP) inhibitor [28]. Therapy with other CETP inhibitors, such as evacetrapib or TA-8995, provided similar effects on the laboratory level [29,30].

Lp(a) is known to mediate calcification and inflammation within stenotic aortic valves by regulating oxidized phospholipids content. The latter are recognized by macrophages scavenger receptors leading to foam cells formation. Therapies targeting apolipoprotein(a) (apo(a)), designed to reduce apo(a) hepatic synthesis with a use of antisense oligonucleotides and small interfering RNA (siRNA), have been shown to decrease Lp(a) levels in double-blinded trials (IONIS-APO(a)Rx_,_ ISIS-APO(a)Rx, and IONIS-APO(a)-LRx) [31,32]. Currently, siRNA-based therapy with olpasiran is tested in a phase II clinical trial (NCT04270760) in patients with atherosclerotic cardiovascular disease. Of note, recent trials ORION-10,11 (Inclisiran for Participants With Atherosclerotic Cardiovascular Disease and Elevated Low-density Lipoprotein Cholesterol) have shown that inclisiran, siRNA-based PCSK-9 inhibitor reduced LDL cholesterol levels by about 50% in patients with atherosclerotic cardiovascular disease and in subjects with genetic hypercholesterolemia (Table 1) [33].

**Figure 1:**
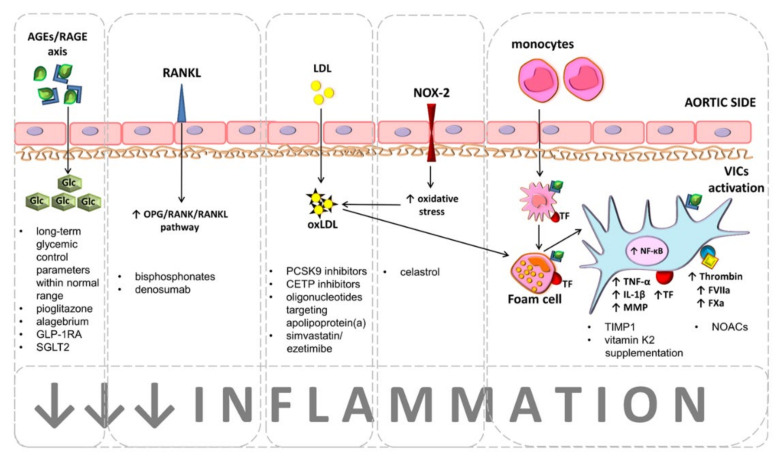
Potential therapeutic targets to retard calcific aortic stenosis (CAS) development or progression.

Adding to the discussion on the role of lipid lowering therapy in CAS, a secondary analysis of the SEAS trial revealed that simvastatin/ezetimibe combination reduced the risk of aortic valve replacement by 60% in patients with mild but not moderate CAS [34]. An ongoing phase 2 trial (NCT03051360) will show whether PCSK9 inhibitors are able to reduce valvular calcification in CAS patients at early stages of the disease. In conclusion, lipid-lowering therapies are currently a promising research topic in CAS development/progression (Figure 1), but available data is insufficient to support any hypolipemic therapy for slowing CAS progression at this point.

Hyperglycemia associated with tissue accumulation of advanced glycation end products (AGEs), which are bound by a specific receptor (RAGE) accelerate calcification of aortic valves and disease progression. Maintaining long-term glycemic control parameters, such as glycated hemoglobin and/or fructosamine levels as well as the treatment with antidiabetic drugs, namely pioglitazone, alagebrium, and glucagon-like peptide-1 receptor agonists (GLP-1RA) or sodium-glucose cotransporter-2 inhibitors (SGLT2) are recognized as beneficial for CAS patients with concomitant diabetes mellitus. Bisphosphonates and denosumab are considered as therapeutic option for CAS patients with concomitant osteoporosis. Both agents interact with the osteoprotegerin (OPG)/receptor activator for nuclear factor kappa B (RANK)/RANK ligand (RANKL) axis, leading to increased expression of OPG, preventing RANK activation, which is responsible for osteogenic differentiation of valvular interstitial cells (VICs).

Chronic valvular inflammation driven by lipids accumulation is a main contributor to CAS progression. Proprotein convertase subtilisin/kexin type 9 (PCSK9) inhibitors, cholesteryl ester transfer protein (CETP) inhibitors, antisense oligonucleotides targeting apolipoprotein(a), and simvastatin/ezetimibe combination are currently tested as agents inhibiting CAS development and progression. Accumulated low-density lipoproteins (LDL) within valve leaflets undergo oxidation (oxLDL) by reactive oxygen species (ROS) generated via NADPH oxidase 2 (NOX2). oxLDLs phagocyted by valvular macrophages lead to formation of proinflammatory foam cells. Proinflammatory microenvironment activates VICs by nuclear factor kappa B (NF-κB) pathway. Activated VICs release tumor necrosis factor α (TNF-α), interleukin 1β (IL-1β), and matrix metalloproteinases (MMPs) and express active coagulation factors, such as tissue factor (TF), factor (F)VIIa, FXa, and thrombin (FIIa). Current therapeutic approaches focus on inhibition of both valvular inflammation and coagulation activation. Celastrol, tissue inhibitor of MMPs (TIMP-1), and vitamin K may exert similar effect aimed at prevention of oxidative stress, inflammation, and VICs calcification. Non-vitamin K antagonist oral anticoagulants (NOACs) inhibit inflammation and coagulation, preventing VICs calcification.

### 2.2. Anti-Hypertensive Therapies

Arterial hypertension and CAS commonly coexist, and the incidence of both conditions increases with age, but the causal relationship remains disputable [35]. In patients with CAS, hypertension adds to pathologic left ventricular (LV) hypertrophic remodeling increasing the afterload [36], but a stiff arterial system, seen in long standing hypertension, may also arguably contribute to the degeneration of the valve by alteration of mechanical stress, leading to endothelial injury [37]. Looking at the larger picture, in 3.39 million studied patients, the probability of having hypertension was four times higher if the patient had CAS [38]. Another study showed that the diagnosis of hypertension increases relative risk of CAS by 23% [6], and a large analysis form UK, including 5.4 million patients without known cardiovascular disease, demonstrated that long-standing exposure to high blood pressure increased the risk of developing CAS (each 20 mmHg increase in systolic blood pressure was associated with risk of CAS increased by 41%) [39]. Hypertension is a risk factor for developing AS and, in small studies, has been associated with progression of stenosis and valve calcification [40,41]. A common denominator is likely the neuroendocrine activation, although the molecular mechanisms in combined hypertension and AS are not fully studied. Activation of the sympathetic nervous system and increased expression of angiotensin II and angiotensin-converting enzyme (ACE) have been documented in CAS [42,43]. While the role of renin–angiotensin–aldosterone (RAAS) in myocardial fibrosis and remodeling is best studied [36], experimental studies point to its role in CAS pathobiology. In a murine model, administration of angiotensin II to genetically hyperlipidemic mice induced aortic valve thickening, endothelial disruption and accumulation of myofibroblasts [44]. In vitro studies on porcine aortic valvular myofibroblasts demonstrated that angiotensin II can promote osteoblast-like transformation of VICs [45], and the presence of ACE has been demonstrated in human valves, being upregulated in stenotic valves [42], and colocalized with apolipoprotein B [46].

ACE inhibitors (ACEI) have been studied in CAS mostly for their beneficial effects on hemodynamics and reverse remodeling of myocardium [36,47]. Trandolapril was shown to improve hemodynamic LV load in patients with CAS, when compared with placebo [48], while ramipril was reported to have positive effects on LV hypertrophy regression in moderate-to-severe CAS (Ramipril In Aortic Stenosis—RIAS trial) [49]. More inconsistent results are available for angiotensin receptor blockers (ARB). Candesartan, although well tolerated by patients with severe symptomatic CAS, was not shown to improve LV geometry or function, or symptoms, compared with placebo (The Potential of Candesartan to Retard the Progression of Aortic Stenosis—ROCK-AS trial) [50], while two studies by Côté and co-workers showed that ARBs contribute to reduction of fibrocalcific remodeling of the aortic valves and inflammation [51,52]. In an experimental rabbit model, ramipril was reported to retard the development of CAS [53], but in a study of 211 patients with CAS, ACEI were not shown to slow the disease progression [54]. A Canadian report on 338 subjects with CAS showed that hypertension was associated with faster CAS progression and higher incidence of clinical events, which were abolished by ARBs, but not ACEI [55]. A retrospective analysis by O’Brien and colleagues [56] using computed tomography (CT)-based assessment of valvular calcium found an association between ACEI use and a lower rate of aortic valve calcium accumulation. Overall, RAAS blockade has beneficial effects in CAS patients, as evidenced by an observational study of 2117 CAS patients, of whom one-third received ACEI or ARB [57]. Treatment with ACEI or ARB reduced all-cause mortality and cardiovascular events during a mean follow-up of four years [57]. Of the ongoing trials, ALFA trial (Randomized Trial of Angiotensin Receptor bLocker, Fimasartan, in Aortic Stenosis) was designed to test the influence of fimasartan on hemodynamics in CAS, but the status of the study is unknown (NCT01589380). A study evaluating losartan (NCT03666351) is still recruiting. A new randomized placebo-controlled trial, with expected completion date in 2025 will assess the efficacy of ARBs in slowing down CAS progression and LV remodeling/dysfunction in patients with mild-to-moderate aortic stenosis (Angiotensin Receptor Blockers in Aortic Stenosis—ARBAS study, NCT04913870). Another interesting aspect of CAS treatment with ACEI/ARBs is post-intervention therapy after SAVR or TAVI. A trial evaluating valsartan vs. placebo (Efficacy of Angiotensin Receptor Blocker Following aortic Valve Intervention for Aortic Stenosis—ARISTOTE trial, NCT03315832) initiated postprocedurally after surgery or percutaneous intervention is yet to start recruiting. In summary, while there is little to unequivocally suggest that RAAS blockade could slow CAS progression, ACEI or ARB are suggested as the first line therapy in CAS patients with concomitant hypertension [36].

A completely new role of RAAS system in CAS might have emerged with the onset of the recent Coronavirus disease 2019 (COVID-19) pandemic. The cellular receptor of severe acute respiratory syndrome coronavirus 2 (SARSCoV-2) is the angiotensin-converting enzyme 2 (ACE2), which may link cardiovascular diseases and SARS-CoV-2 susceptibility. Fagyas and colleagues reported four times higher levels of circulating ACE2 levels in patients with severe CAS than in hypertensive controls, which could arguably make them more prone to infection and its complications [58].

### 2.3. CAS Patients with Kidney Disorders

Chronic kidney disease (CKD) is associated with the risk of CAS development, both in non-dialysis dependent individuals [59] and, more prominently, in those requiring dialysis [60]. Results from the CIRC study (Circulation Improving Resuscitation Care) showed that the estimated glomerular filtration rate (eGFR) is associated in a dose-dependent manner with aortic valve calcification (measured in CT) in patients with CKD, and this association was independent of cardiovascular risk factors [61]. Multiple contributors have been postulated: hyperphosphatemia, calcium–phosphate product, parathyroid hormone, and β2-microglobulin [60,62]. Vascular calcification was shown to be associated with low vitamin D levels [63], and CAS progression has been associated with secondary hyperparathyroidism in elderly patients with CKD and low vitamin D levels in COFRASA study (Aortic Stenosis in Elderly: Determinant of Progression) [64]. Another postulated contributor could be amyloid deposition within the valve [65].

In vitro, an increase in phosphate concentration is responsible for VICs activation and osteoblast-like transformation, related to increased expression of osteogenic factors, such as Runx2, Osterix (Osx), and differentiation factors such as BMP-2 [66]. Additionally, parathyroid hormone elevation is associated with increased aortic valve calcification [67] and more rapid progression of CAS [68]. Inhibitors of ectopic soft tissue calcification, fetuin A and matrix Gla protein (MGP), play a protective role against valvular mineralization [69]. Fetuin A forms ‘calciprotein particles’, calcium and phosphate containing colloidal complexes in serum, and low circulating levels of fetuin A have been reported to be associated with rapid CAS progression both in the general population [70] and dialysis patients [71]. MGP is a protein expressed primarily in the cartilage, heart, and vessels, acting as an inhibitor of cardiovascular calcification [69,72]. Its active form, the ‘carboxylated MGP’ (cMGP) is formed by glutamate residues carboxylation with vitamin K. cMGP reduces calcium precipitation and osteogenic transition of VICs [73], and its levels are lower in CKD than in healthy individuals [74]. Data on the expression and metabolism of MGP within the aortic valve are unfortunately limited.

Fibroblast growth factor-23 (FGF-23) is a phosphaturic hormone produced in the bone, controlling the inorganic phosphate excretion, while Klotho is a protein responsible for the FGF-23 kidney-specific action [75]. FGF-23 participates in activated vitamin D circulating levels reduction [76], and an association between increased FGF-23 and reduced Klotho with vascular calcification and aortic valve calcification in CKD patients was previously reported [77,78,79]. A study from China reported that human VICs express Klotho, and Klotho suppresses the pro-osteogenic effect of high phosphate on VICs [80]. Its potential therapeutic role remains to be studied.

Inhibition of acetylation of histones 3 and 4 could be an interesting potential therapeutic target. Gu et al. reported porcine VICs in vitro experiments that osteoblastic transformation could be attenuated by the histone acetyltransferase inhibitor C646 [81]. In this experimental study, C646 decreased the acetylation of histones 3 and 4 induced by the high-calcium/high-phosphate treatment, while the histone deacetylase inhibitor suberoylanilide hydroxamic acid promoted VICs calcification [81].

Furthermore, chronic inflammatory state and uremic toxins are postulated to stimulate the inflammatory state within the valve in chronically dialyzed patients [69].

Data on delaying the valvular calcification and hence progression of CAS in CKD are sparse. The ADVANCE study (Study to Evaluate Cinacalcet Plus Low Dose Vitamin D on Vascular Calcification in Subjects With Chronic Kidney Disease Receiving Hemodialysis) tested the use cinacalcet (a calcimimetic agent) plus low-dose vitamin D on vascular and valvular calcification in patients CKD on dialysis [82], showing a significant retardation of the progression of aortic valve calcification in CT, but it is still unknown whether this would protect against CAS progression. The EVOLVE study (Evaluation Of Cinacalcet Hydrochloride (HCl) Therapy to Lower CardioVascular Events) did not show a reduction of risk of death or major cardiovascular events in patients with moderate-to-severe secondary hyperparathyroidism who were undergoing dialysis and treated with cinacalcet [83]. Further studies are needed in this high-risk cohort.

### 2.4. Anti-Diabetic Therapies

Clinical interactions between DM and AS progression have been shown previously, however with contradictive conclusions. In AS patients, concomitant DM was associated with higher annual progression in peak systolic gradient [84] and increased risk of valvular calcification [85]. Kamalesh et al. [86] have found faster disease progression in diabetics, but only in patients with moderate AS and concomitant DM compared to non-diabetics. Studies performed on large cohorts confirmed that DM is associated with markedly increased risk for CAS development, ranging from 34 to 49% [87,88]. Furthermore, DM has been identified as an independent risk factor of CAS development by Martinsson [89]. On the contrary, Testuz et al. [90] reported no associations between metabolic syndrome or diabetes and progression of AS in a study prospectively following 200 patients with those conditions. Taken together, while the available data suggest a relationship between DM and CAS development, no treatment recommendations can be made to modulate CAS progression by means of DM treatment. If one takes number of experimental studies into focus, some of them suggest that DM influences valvular calcification. It has been shown that valves obtained from diabetic patients with severe CAS have higher expression of C-reactive protein (CRP), which was associated with valvular expression of tissue factor (TF), the major coagulation trigger in vivo [91] and were characterized by 6.6-fold increased accumulation of advanced glycoxidation end products (AGEs), when compared to non-diabetics [92]. AGEs, whose formation is strictly associated with hyperglycemia, can impair valvular leaflet structure through cross-linking of matrix proteins, and affect multiple cellular processes via interaction with AGEs surface receptor (RAGE) [92]. Interestingly, both clinical and molecular relevance of AGEs in CAS seems to be evident. On the one hand, accumulation of both valvular and plasma AGEs correlated with severity of CAS measured echocardiographically [92]. On the other hand, AGEs accumulation within human aortic stenotic valves was associated with enhanced valvular inflammation, measured as an expression of nuclear factor κB (NF-κB), coagulation activation, as evidenced by increased expression of factor (FII) and activated FX, and calcification, evidenced by higher valvular amounts of BMP-2 [92]. Similar observations have been reported for rabbit and murine models of CAS [93,94].

Of note, no associations have been observed between valvular inflammation and calcification and fasting glucose concentrations in CAS patients with DM, while inflammation and calcification did correlate with long-term glycemic control parameters, such as HbA1c and fructosamine [92,95].

Growing body of evidence supports the notion that in CAS patients, poorly controlled DM enhances valvular oxidative stress, inflammation, and coagulation activation, which may, arguably, accelerate AS progression. Thus, based on the available data, one might speculate that in CAS patients with DM, maintaining good long-term glycemic control may be of major importance for AS progression retardation. Clinical trials are urgently needed to verify this exciting hypothesis.

Currently, drugs targeting AGEs-RAGE axis inhibition, such as pioglitazone or alagebrium (ALT-711), novel antihyperglycemic agents, namely glucagon-like peptide-1 receptor agonists (GLP-1RA; liraglutide, luraglutide, and semaglutide), and sodium-glucose cotransporter-2 inhibitors (SGLT2; empagliflozin, canagliflozin, dapagliflozin, and ertugliflozin) have been tested in studies involving both human and animals to reduce cardiovascular complications, including AS, in DM [96]. Very recently, it has been shown on mouse and rabbit models that evogliptin, one of the selective dipeptidyl peptidase-4 inhibitors, decreased the expression of proinflammatory cytokines, valvular fibrosis, and calcification of aortic valves [97]. Despite the fact that AGEs-RAGE axis inhibition seems to be a promising therapeutic target (Figure 1), no data is available to prove whether inhibition of the AGEs-RAGE axis has a cardioprotective effect in DM patients. Another hindrance is the fact that to date there are no clinically validated tests to measure AGEs and/or tissue concentrations.

Since currently the only therapeutic options for AS are SAVR or TAVI, it is interesting to look at procedural outcomes in diabetic patients [98,99,100,101,102,103]. A large retrospective study showed that DM patients, compared to non-DM individuals, did not have worse short-term outcomes after TAVI or SAVR [95]. Available evidence shows no differences in 30 day or 1-year all-cause mortality between DM and non-DM patients undergoing TAVI [99,100,101].

On the other hand, according to a substudy of the PARTNER trial (The Placement of Aortic Transcatheter Valves), one-year all-cause mortality was lower in the DM group after TAVI, compared to SAVR [102]. Similarly, Ando et al. [103] showed that all-cause mortality in DM patients after TAVI was about 22% lower compared to this observed in the SAVR group. The decision between TAVI and SAVR is complex and involves many patient-related aspects; current guidelines do not suggest taking DM into consideration when making this decision between surgical or percutaneous approach [2]. Certainly, however, DM adds to the overall surgical risk in patients undergoing SAVR, and is included in risk scores, such as Society of Thoracic Surgeons Predicted Operative Mortality or EuroSCORE II.

### 2.5. Therapies Targeting Calcification

The best suited imaging modality for assessment of aortic valve leaflet calcification in clinical practice is CT, and the degree of valvular calcification is directly associated with CAS severity and survival [104,105,106]. Reduced bone mineral density has been moderately but independently associated with vascular calcification in the MESA substudy (the Multi-Ethnic Study of Atherosclerosis) [107]. However, to date the mechanism of this complex relationship has not been fully understood. Osteoporosis is treated with bisphosphonates, which inhibit bone resorption, and a few small retrospective studies have demonstrated that bisphosphonate use was associated with slower progression of CAS [108,109]. In another retrospective analysis of females older than 60 years with mild-to-moderate CAS, bisphosphonates had no significant impact on both hemodynamic and clinical progression of CAS [110]. Preclinical study with VICs isolated from porcine hearts demonstrated that denosumab, designed to bind receptor activator for NF-κB ligand (RANKL), inhibited calcification with no toxic effect [111]. Interestingly, very recently Pawade et al. [112] published results from a double-blind controlled trial—SALTIRE2 (Study Investigating the Effect of Drugs Used to Treat Osteoporosis on the Progression of Calcific Aortic Stenosis, NCT02132026), in which RANKL inhibitors (denosumab or alendronic acid) were ineffective in inhibition of aortic valve calcification, or even disease progression. Of note, the majority (85%) of the trial population was diagnosed with mild or moderate asymptomatic CAS [112]. Important addition to the topic is a study by Sikura et al. [113], which revealed that hydrogen sulfide (H_2_S) inhibits both in vitro inflammation and calcification in human VICs, and calcification of aortic valves in ApoE deficient mouse via Runx2-regulated NF-κB activation. It is tempting to speculate that Runx2 inhibition is a potential therapeutic strategy, which could retard or inhibit valvular calcification. This needs to be verified by further research.

### 2.6. Inflammation Related Targets

Inflammation is the main driving force of valvular fibrosis and calcification. Valvular inflammation is initiated by overexpression of the NADPH oxidase 2 (NOX2), which is the pivotal cellular producer of reactive oxygen species (ROS) and is under control of the NF-κB pathway, leading to activation of proinflammatory, and coagulation-, and calcification-related genes [114,115,116]. Moreover, NOX2 has been associated with VICs calcification. NOX2 inhibition by celastrol was shown to prevent VICs calcification, ROS generation as well as valve fibrosis and LV remodeling in a rabbit model of CAS [117].

Activation of VICs leads to increased degradation of the ECM and enhanced expression of both matrix metalloproteinases (MMPs) and tissue inhibitors of MMPs (TIMPs). MMPs, as zinc- and calcium-dependent endopeptidases, play a central role in many physiological and pathological processes, including valvular calcification [118,119,120,121]. The expression of MMPs may be regulated, among other factors, by NF-κB [121,122] and pro-inflammatory cytokines [119,120,121]. Within the leaflets of a stenotic aortic valve, the presence of several MMPs (such as MMP 1-3, MMP-7, MMP-9, MMP-10, and MMP-12) has been demonstrated [119,123,124,125]. MMPs in in vitro VICs cultures increased the levels of inflammatory, fibrotic and calcification markers, while the addition of a TIMP-1 or monoclonal antibody against MMP-10, prevented inflammation and cellular calcification [125]. Recently, it has been demonstrated in patients with CAS that plasma MMP-28 level is associated with the disease severity in echocardiographic measurements [126]. MMPs are hence emerging as potential therapeutic targets for retardation of the aortic valve calcification and CAS progression, yet this territory remains uncharted. Experimental and clinical studies testing the efficacy of antibodies against MMP-10 might open new therapeutic options for patients with mild-to-moderate CAS (Figure 1).

### 2.7. Coagulation Activation Related Targets

It has been shown that the stenotic aortic valves exhibit the expression of several coagulation factors, such as TF, prothrombin, factor (F) Xa, fibrin, and FXIII [127,128,129,130,131]. The expression of coagulation factors correlated with disease severity reflected by transvalvular pressure gradients and/or aortic valve area [127,128,129,130,131]. Breyne and co-workers [128], demonstrated the co-localization of TF and thrombin with osteopontin within human stenotic aortic valves, suggesting that coagulation activation interacts with the processes of valvular calcification. Similar results were obtained for VICs cultures stimulated with proinflammatory factors, which expressed TF, FVII, FXa, thrombin, and protease-activated receptors (PAR1 and PAR2) [131]. Growing body of evidence indicates that inadequate proteolytic activation of PARs may influence several cellular processes, including proliferation, migration and apoptosis, contributing to the pathological fibrotic remodeling, inflammation, and coagulation [132,133]. These findings support the concept that inflammation and coagulation are tightly linked, and both involved in valvular fibro-calcification.

It has been demonstrated that valvular calcification can be influenced by anticoagulant therapy [134,135,136,137]. Warfarin, a vitamin K antagonist (VKA), has been associated with vascular calcification through the inhibition of gamma-carboxylation of coagulation factors and other vitamin K-dependent proteins, such as MGP, a potent inhibitor of calcification [134]. It has been demonstrated in patients with symptomatic severe CAS that plasma level of undercarboxylated MGP (ucMGP) was associated with all-cause mortality and indices of heart failure [138]. Koos et al. [135] reported in a small case–control study that long-term warfarin treatment (mean anticoagulant duration 88 ± 113 months) in patients with CAS was associated with 125% increased aortic valve calcification score, compared with patients not taking warfarin. Moreover, they have found 39% lower serum MGP levels in CAS patients on warfarin, compared with the control group [136]. Therefore, supplementation of vitamin K has been proposed as a novel therapeutic strategy to reduce calcification of both vessels and valves. Vitamin K1 and K2 have been shown to decrease the levels of proinflammatory cytokines and adhesive molecules, while increasing the LDL receptor expression, which leads to a reduction of lipid levels [139,140,141]. Slower calcification of aortic valves has been associated with supplementation with vitamin K1 [142]. Currently, patients without clinically significant CAS or with mild-to-moderate CAS and bicuspid aortic valve taking vitamin K2 are studied [143,144].

Non-VKA oral anticoagulants (NOACs) deserve a comment. Recently, Di Lullo et al. [145] have reported that the use of rivaroxaban was associated with reduced valvular calcium deposition and CAS progression as well as with lower serum CRP levels compared to warfarin in patients with chronic kidney disease. Several animal and in vitro studies indicated that NOACs, namely dabigatran (a direct thrombin inhibitor), rivaroxaban and apixaban (FXa inhibitors), are able to exert not only anticoagulant effects, but also have anti- inflammatory properties [131,145,146,147,148,149,150,151]. Studies in ApoE deficient mice revealed that dabigatran and rivaroxaban can attenuate atherosclerotic plaque progression by reduction of lipid deposition and collagen content, macrophage accumulation, ROS production, and expression of inflammatory mediators [146,147,148,149,150,151]. It has been also demonstrated that rivaroxaban can inhibit the expression of NF-κB pathway and prevent upregulation of TF, plasminogen activator inhibitor-1 (PAI-1), and PARs expression in atrial tissue cultures [151]. Moreover, VICs treated with rivaroxaban or dabigatran at therapeutic concentrations inhibited expression of coagulation factors and PARs, as well as proinflammatory cytokines, MMPs, and calcification markers on both protein and mRNA levels [131]. Interestingly, the COMPASS trial (Rivaroxaban for the Prevention of Major Cardiovascular Events in Coronary or Peripheral Artery Disease), involving over 27,000 patients, has shown a reduced rate of the cardiovascular death, stroke, or myocardial infarction (4.1% vs. 5.4%, HR = 0.76, 95% CI 0.66–0.86, *p* < 0.001) in patients taking low dose of rivaroxaban (2.5 mg twice daily) with aspirin (100 mg once daily) as secondary prevention, compared to aspirin alone [152].

Observational and experimental studies suggest that NOACs may influence valvular fibro-calcification remodeling and inflammation and could serve as a therapeutic strategy to reduce CAS progression, while having overall beneficial cardiovascular effects, at least in patients requiring anticoagulant therapy (Figure 1).

### 2.8. Inhibition of Other Calcification-Related Molecular Pathways

Ectonucleotide pyrophosphatase/phosphodiesterase 1 (ENPP1) hydrolyzing extracellular ATP to generate pyrophosphate is a promising target to inhibit valvular calcification [153]. ENPP1 has been shown to be overexpressed in calcific aortic valves and to promote mineralization of VICs [154]. Therefore, ENPP1 inhibitors are considered as the potential agents able to retard or inhibit valvular calcification. ARL67156, an inhibitor of ectonucleotidases, prevented CAS development in a rat model of aortic stenosis [155]. Unfortunately, to the best of our knowledge there have been no clinical trials to confirm a beneficial effect of ENPP1 inhibitors in CAS patients. It is also uncertain what other clinical effects would result from a such therapy.

Recently, a Cadherin-11-blocking antibody (SYN0012) has been suggested as a novel pharmacological strategy to prevent CAS progression, and cadherin-11 knock-down mice did not develop CAS [156]. However, an inhibition of this molecular pathway has not been studied in human subjects and needs further investigation.

Nitric oxide (NO), a natural vasodilator, is produced by VECs to prevent matrix calcification and VICs activation [157]. Under pathological conditions, the expression of valvular endothelial NO synthase (eNOS) is downregulated, leading to increased oxidative stress, inflammation, and valvular calcification [158]. It has been shown that L-Arginine, the main precursor of NO, prevents osteogenic differentiation of VICs [159]. Very recently, Majumdar et al. [160] have discovered a new molecular mechanism where NO regulates NOTCH1 signaling pathway in CAS via ubiquitin-proteasome system. Moreover, investigators revealed that S-nitrosylation, a NO-dependent posttranslational modification, inhibits calcification of porcine VICs in in vitro experiments [160]. Interestingly, van Driel et al. [161] using mass spectrometry identified 30 serum metabolites that distinguished patients with AS from healthy individuals, 17 of which were related to NO metabolism. One may consider the NO pathway modulation as another possible target for CAS progression inhibition; however, the data remain sparse and there are no clinical studies investigating this pathway.

## 3. Future Perspectives

Several promising targets based on molecular pathways involved in valvular calcification are currently investigated, and the knowledge on molecular pathobiology of CAS is growing rapidly. Since most of studies investigating drugs targeting calcification-related molecular pathways have been performed in vitro, animal studies are needed to provide the proof of concept for efficacy with minimal side effects (Table 2). Further, clinical randomized trials will be needed to provide compelling evidence that personalized inhibition of particular molecular pathways is a therapeutic option for patients with CAS.

## 4. Conclusions

To date, the current standard of care for patients with CAS remains surgical or percutaneous intervention at the very late, symptomatic stage of the disease natural progression and there is no approved pharmacological treatment to prevent or retard CAS progression. Lipid-lowering therapies have been shown to exert beneficial effects, preventing CAS progression in patients with mild-to-moderate AS; however, this does not include statins. In the future, personalized pharmacological treatment orchestrated to take cognizance of concomitant diseases may retard CAS progression or minimize the risk of adverse complications. Introduction of pharmacological retardation of AS by targeting calcification-related molecular pathways would revolutionize AS treatment but it probably requires many years of clinical studies.

## Figures and Tables

**Table 1:** Clinical trials testing therapies targeting hypercholesterolemia in patients with cardiovascular disease.

Author, Year of Publication, and Trial Acronym	Study Type	Study Design	Main Findings
Bergmark et al., 2020, the FOURIER trial [26]	randomized clinical trial	13,784 atherosclerotic vascular disease patients taking PCSK9 inhibitor evolocumab (mean age, 63 ± 9 years) and 13,784 taking placebo (mean age, 62 ± 9 years), including 63 patients who developed CAS in the full cohort	Long-term therapy with evolocumab (>1 year) may reduce AS events (HR 0.48; 95% CI, 0.25–0.93)
HPS3/TIMI55-REVEAL Collaborative Group; Bowman et al., 2017, the REVEAL trial [28]	randomized clinical trial	15,225 atherosclerotic vascular disease patients assigned to receive CEPT inhibitor anacetrapib (mean age, 67.8 ± 8 years) and 15,224 patients taking placebo (mean age, 67.8 ± 8 years)	Long-term therapy with anacetrapib was associated with reduced Lp(a) (mean level, 43 vs. 58 nmol/L) and LDL cholesterol levels (mean level, 38 vs. 64 mg/dL), and increased HDL cholesterol levels (mean level, 85 vs. 42 mg/dL)
Nicholls et al., 2016 [29]	randomized clinical trial	398 patients with elevated LDL cholesterol or low HDL cholesterol, including 118 patients taking evacetrapib (mean age, 58.6 ± 10.8 years) for 12 weeks as monotherapy or in combination with statins compared to placebo (mean age, 58.9 ± 11.4 years)	Short-term therapy with evacetrapib reduced Lp(a) levels up to 40% and LDL cholesterol up to 54%
Hovingh et al., 2015, the TULIP trial [30]	randomized clinical trial	149 patients with mild dyslipidemia taking CETP inhibitor TA-8995 as monotherapy, 151 patients taking TA-8995 in combination with statins for 12 weeks, and 37 patients receiving placebo	Short term therapy with TA-8995 reduced the concentrations of LDL cholesterol up to 68.2% and increased the levels of HDL cholesterol up to 179%
Tsimikas et al., 2015 [31]	randomized clinical trial	47 healthy volunteers (mean age 35 ± 16.9) with BMI < 32 kg/m^2^ and Lp(a) ≥ 25 nmol/l taking antisense oligonucleotide ISIS-APO(a)_Rx_ for 4 weeks	ISIS-APO(a)_Rx_ reduced Lp(a) concentrations in a dose-dependent manner up to 77.8%
Viney et al., 2016 [32]	randomized clinical trial	64 healthy volunteers (mean age 58 ± 8 years) with elevated Lp(a) taking antisense oligonucleotide IONIS-APO(a)_Rx_ and 58 individuals (mean age 56 ± 5 years) taking IONIS-APO(a)-LRx or placebo for 12 weeks	IONIS-APO(a)_Rx_ reduced Lp(a) concentrations up to 71.6% and IONIS-APO(a)-LRx up to 92%
Ray et al., 2020, the ORION trial [33]	randomized clinical trial	1591 patients at high risk for cardiovascular disease and increased LDL cholesterol levels taking PCSK9 inhibitor inclisiran and 1587 patients taking placebo for 18 months	Inclisiran reduced LDL cholesterol levels approximately by 50%
Greve et al., 2019, secondary analysis of the SEAS trail [34]	randomized clinical trial	1687 asymptomatic patients with mild to moderate CAS taking simvastatin/ezetimibe combination vs. placebo for median time of 4.3 years	Simvastatin in combination with ezetimibe reduced the rate of aortic valve replacement in patients with mild AS (HR 0.4; 95% CI, 0.2–0.9)

**Table 2:** The most promising future perspectives regarding treatment of aortic stenosis.

	Therapy	Target of Therapy	Risk of Therapy
**Lipid-lowering therapies**	PCSK9 inhibitors (including siRNA)	Reduction in Lp(a) and LDL cholesterol, increased HDL levels associated with reduced valvular inflammation leading to reduced calcium accumulation.	PCSK9 inhibitors may increase the risk of neurocognitive effects, new onset DM or statin-associated muscle symptoms or other adverse events [162].
	CETP inhibitors	Reduction in the concentrations of Lp(a), LDL cholesterol, and other lipoproteins, increased HDL levels resulting in decreased valvular inflammation.	CETP inhibitors activate the renin–angiotensin system increasing blood pressure with its attendant cardiovascular risks, thus in patients with hypertension and CAS further investigations of a possible interaction between the use of antihypertensive drugs and CETP inhibitors are needed [163].
	Antisense therapy: IONIS-APO(a)Rx, IONIS-APO(a)-LRx	Reduction in Lp(a) concentrations leading to decreased valvular inflammation.	No serious side effects [32], however, long-term follow-up is needed to confirm this observation.
**Therapies used in patients with CKD**	HAT inhibitor: C646	C646 attenuated aortic valve calcification both in vitro and in vivo.	HAT inhibition is not selective and it suppresses osteoblast-related gene expression leading to decreased osteogenic differentiation.
	Calcimimetic: cinacalcet + low dose vitamin D	Cinacalcet + low-dose vitamin D sterols attenuated vascular and cardiac valve calcification.	Adverse gastrointestinal effects associated with cinacalcet treatment occurring in about 10% of patients [82,83].
**Inflammation related targets**	NF-κB inhibition	NF-κB inhibition prevented VICs calcification in cultures treated with high concentrations of glucose.	A better understanding of the molecular regulation that determines the point of conversion of NF-κB responses from protective to damaging effects is needed for therapeutic intervention in humans.
	Glucagon-like peptide-1 receptor agonists	Decreased valvular inflammation, cytokine expression, fibrosis, and calcification in a CAS animal model.	Allergic reactions, upper respiratory tract infections, and urinary tract infection have been reported [164]. To date, not investigated in CAS patients.
	Precursor of NO: L-Arginine	L-Arginine inhibited induced VICs calcification.	Known to worse asthma symptoms [165]. To date, not investigates in CAS patients.
	NOX2 inhibitors	Celastrol prevented VICs calcification, ROS generation, valve fibrosis and left ventricular remodeling in a rabbit model of CAS.	Non-selective substances, which theoretically may exert pro-inflammatory and autoimmune effects [166]. A proof of concept for efficacy with minimal side effects is needed for the acceptance of NOX2 inhibitors as therapeutic agents in CAS patients.
	Tissue inhibitor of metalloproteinases:TIMPs	TIMP-1 prevented VICs inflammation and calcification.	Non-selective substances, the next generation of MMPs inhibitors must be selective against MMPs, such as MMP-3, -9, -10 or -12.
**Therapies targeting coagulation**	NOACs	Rivaroxaban and dabigatran inhibited VICs calcification and inflammation.	Bleeding, anemia. To date, not investigated in CAS patients with regard to CAS progression.

## Data Availability

Data sharing is not applicable to this article.
